# Leaflet Perforation Using Cor-Knot in Minimally Invasive Aortic Valve Replacement

**DOI:** 10.1016/j.atssr.2024.01.003

**Published:** 2024-02-06

**Authors:** Takayuki Kawamura, Ken Chen, Yosuke Mukae, Kenta Zaikokuji, Tomohiro Iwakura, Tomoki Shimokawa

**Affiliations:** 1 Department of Cardiovascular Surgery, Adult, Sakakibara Heart Institute, Tokyo, Japan

## Abstract

A 38-year-old woman underwent minimally invasive aortic valve replacement with a 21-mm Inspiris aortic valve and Cor-Knot for type 0 bicuspid valve and severe aortic stenosis. Postoperative transthoracic echocardiography was uneventful. Four months later, she experienced shortness of breath. Transthoracic echocardiography revealed moderate to severe aortic valve regurgitation, and prosthetic valve dysfunction was suspected. Aortic valve reoperation through a median sternotomy was performed. The bioprosthetic valve was perforated at the noncoronary cusp valve site, probably because of prolonged exposure to the Cor-Knot. When Cor-Knot is used in a narrow space, a perpendicular apposition should always be maintained between the sewing ring and the Cor-Knot tips.

The Cor-Knot (LSI Solutions) automated fastener has been reported to reduce aortic cross-clamp time, cardiopulmonary pump time, and operative times in past studies.^1^ The ligatures are described as much stronger and more uniform than those tied by hand or with a knot pusher.^2^ The occurrence of postoperative paravalvular leak is lower in patients treated with the Cor-Knot under minimally invasive cardiac surgery aortic valve replacement (MICS-AVR) than in those not treated with the Cor-Knot.^4^ In this report, we describe a case of postoperative aortic regurgitation due to perforation of the valve by a clip after implantation in a patient who underwent MICS-AVR with a Cor-Knot for a type 0 bicuspid valve. Perforation may be related to the application of the automated fastener device (Cor-Knot) through the right thoracotomy approach.

A 38-year-old woman with a past history of infantile asthma presented with shortness of breath. She had undergone MICS-AVR with a 21-mm Inspiris aortic valve (Edwards Lifesciences) for type 0 bicuspid and severe aortic valve stenosis.

We performed minimally invasive aortic valve replacement for a type 0 non-raphe bicuspid aortic valve (Figure 1A). A right lateral mini-thoracotomy was performed through the third intercostal space. A videoscope was inserted through the port into the third intercostal space along the midaxillary line. A cardiopulmonary bypass was performed through the right femoral artery and vein, and aortotomy was performed 2.5 cm above the right coronary artery. The aortic valve was bicuspid with severe calcification. After valve removal, replacement was performed with a 21-mm Inspiris aortic valve. This valve was implanted in the supra-annular position with 13 single sutures using a Cor-Knot automated fastener (Figure 1B). The patient was easily weaned off cardiopulmonary bypass, and protamine was administered systemically.

**Figure 1:**
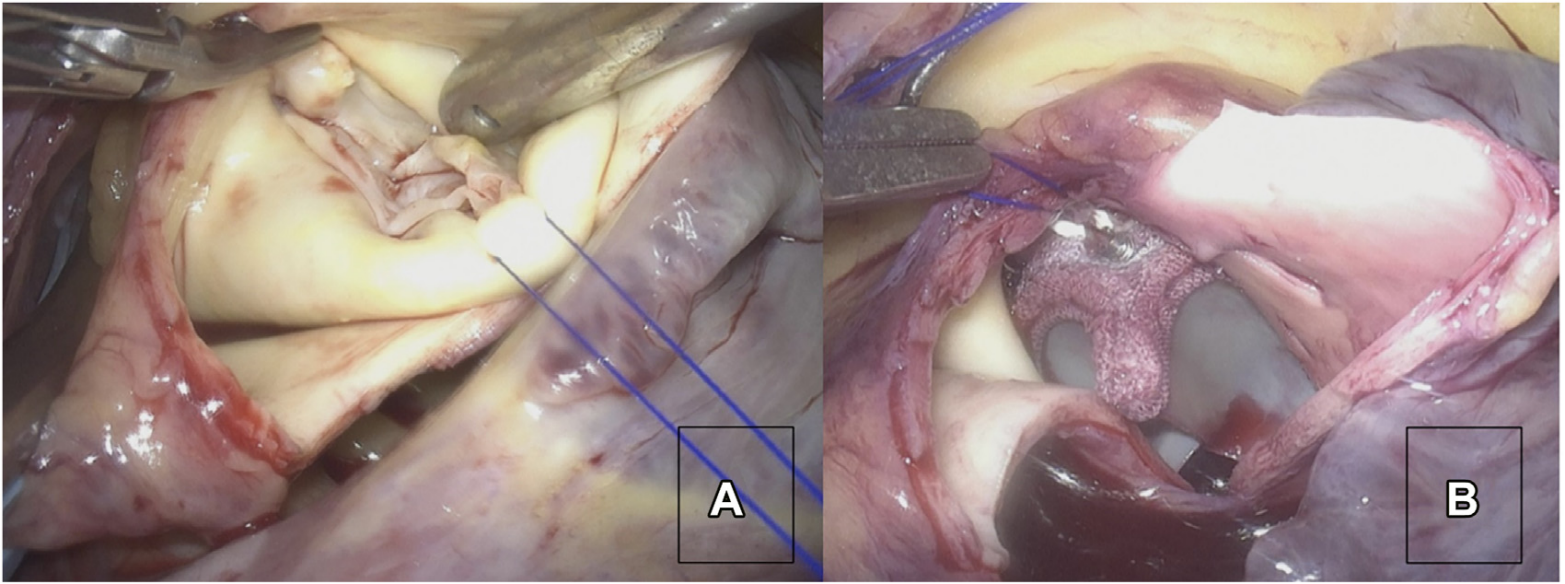
First intraoperative findings. (A) Intraoperative view of bicuspid aortic valve type 0. (B) Postoperative view of the aortic prosthetic valve. A 21-mm Inspiris bioprosthetic valve was implanted into the aortic valve position.

Postoperative echocardiography revealed no obvious complications and showed no aortic regurgitation; aortic valve velocity was 2.0 m/s, and mean pressure gradient was 7.9 mm Hg. The patient was discharged on postoperative day (POD) 11. Four months later, the patient experienced shortness of breath on exertion and visited our clinic. Echocardiography revealed moderate to severe aortic valve regurgitation, and prosthetic valve dysfunction was suspected. The patient then underwent reoperation.

The preoperative laboratory values were as follows: white blood cell count, 7200 cells/μL; hemoglobin concentration, 11.4 g/dL; platelet count, 240,000 cells/μL; prothrombin time–international normalized ratio, 1.12; and creatinine concentration, 0.72 mg/dL. Computed tomography revealed the ascending aorta to be 27 mm with no calcification. The angle between the valve and aorta was 42 degrees, and the following measurements were obtained: annulus, 19.7 mm; aortic valve diameter, 19.7 mm (using perimeter), 19.6 mm (using area).

Transthoracic echocardiography revealed the following: left atrium diameter, 29 mm; left ventricular diameter at end diastole/systole, 38/26 mm; ejection fraction, 64.1%, asynergy; aortic regurgitation, moderate; mitral regurgitation, trivial; tricuspid regurgitation, trivial (right ventricular systolic pressure, 29 mm Hg); aortic valve maximum velocity, 3.1 m/s; aortic valve area, 1.1 cm^2^; maximum/mean pressure gradient, 37.6/21.1 mm Hg; and moderate to severe regurgitation blowing upward at the 4- to 6-o’clock position.

Furthermore, transesophageal echocardiography indicated moderate aortic regurgitation after valve replacement. The valve leaflet at the 9- to 12-o'clock position in the minor axis (valve leaflet shifted toward the left ventricle) was deformed and deviated; a regurgitant valve orifice was observed in the center of the valve leaflet at the same site and showed moderate transvalvular regurgitation. The regurgitant jet blew in the direction of apex deviation at the same site. Computed tomography showed that the prosthetic valve in the aortic position appeared to be inserted along a deviated axis. The left side of the prosthetic valve was shifted toward the left ventricle and the right side toward the aorta (Figure 2).

**Figure 2:**
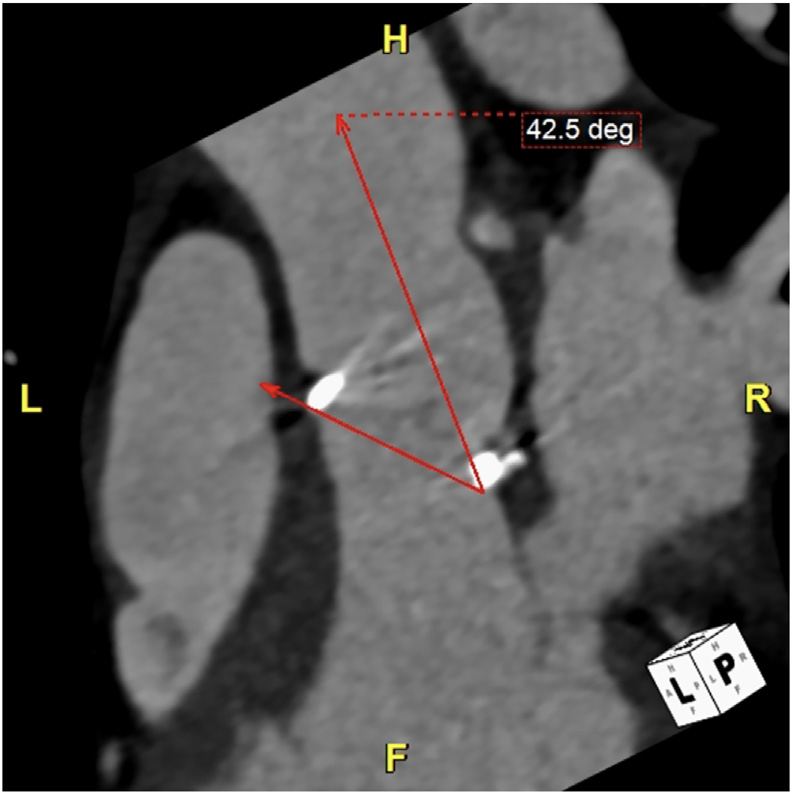
Computed tomography scan showing the angle between the aorta and valve to be 42.5 degrees. (F, foot.; H, head; L, left; R, right.)

Aortic valve reoperation was performed through median sternotomy. Cardiopulmonary bypass were performed on the ascending aorta and right atrium. There was moderate adhesion on the pericardial sac. The Inspiris valve was implanted slightly higher on the right coronary cusp side, and the valve itself was degenerating (Figure 3A). The noncoronary cusp valve was perforated because of the stress experienced by the Cor-Knot (Figure 3B). The 13 Cor-Knot stitches were removed, and the Inspiris valve was explanted. A 19-mm St Jude Medical Regent (Abbott Cardiovascular) mechanical valve was implanted by 13 stitches with single-knot suture using hand tie. Aorta cross-clamp time was 93 minutes, and cardiopulmonary pump time was 121 minutes.

**Figure 3:**
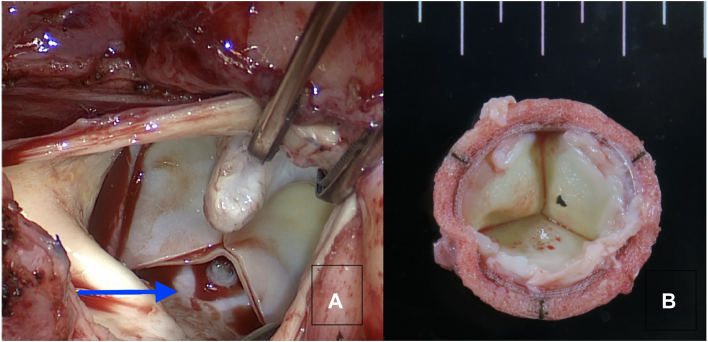
Second intraoperative findings. (A) Intra operative view of punctate defects (arrow) in the pericardial leaflets shows deviated angle of the valve and aorta. (B) Explanted aortic valve with perforation caused by the Cor-Knot.

Postoperatively, the patient was transferred to the intensive care unit and was weaned off the ventilator for 5 hours. On POD 6, transthoracic echocardiography showed good cardiac contraction: aortic valve area, 1.8 m^2^; Vmax, 1.9 m/s; peak pressure gradient, 15.2 mm Hg; and mean pressure gradient, 7.7 mm Hg. No paravalvular leakage was observed. The patient was discharged on POD 16. Echocardiography performed 1 year postoperatively showed no mechanical valve replacement complications.

## Comment

Previous reports have shown that in MICS-AVR, the use of Cor-Knot reduces cross-clamping and cardiopulmonary bypass times and does not increase perioperative morbidity and mortality.^1^^-^^3^ There have been reports of postoperative aortic and mitral regurgitations due to perforation of the titanium clip after Cor-Knot implantation, which is attributed to the clip’s being driven in with the distal tips facing the valve leaflet.^4^, ^5^, ^6^, ^7^ Salas De Armas and coworkers^8^ reported aortic dissection from the distal tip of the device shaft.

When clips are implanted, they should always be inserted such that they face outward toward the center of the valve. In our case, the Cor-Knot was inserted circumferentially toward the lateral side of the valve; however, when the valve ring was threaded, the leaflet on the noncoronary cusp side was positioned more toward the left ventricle, and the opposing right and left coronary cusp commissures were positioned toward the aorta. The displacement of the angle between the valve ring and aorta caused the tip of the Cor-Knot on the noncoronary cusp side to be oriented toward the valve leaflet.

Correcting the Cor-Knot implantation depends on the angle of deployment of the Cor-Knot. The angle is deflected inward toward the valve ring, increasing the risk of physical contact between the Cor-Knot tip and the valve leaflet. In this case, the crownless valve was dissected such that it deviated toward the left ventricular outflow tract at the left-right fusion leaflet. The valve ring is in contact with the valve. This may have caused postoperative perforation owing to a change in valve durability.

In our institution, Cor-Knot is routinely used during MICS-AVR. However, in cases involving a narrow valve ring or anatomic blind spot, Cor-Knot may be placed at an inappropriate angle, as demonstrated here, and therefore should not be employed. When anatomic limitations are not present, Cor-Knot proves to be beneficial as it simplifies the surgical procedure.

In conclusion, our case demonstrated that using a Cor-Knot in the MICS approach for type 0 bicuspid valve may limit visualization of the sewing site. Maintaining a perpendicular apposition between the sewing ring and the Cor-Knot tips is crucial.
